# Critical diaphragm failure in sudden infant death syndrome

**DOI:** 10.3109/03009734.2010.548011

**Published:** 2011-04-12

**Authors:** Pontus Max Axel Siren, Matti Juhani Siren

**Affiliations:** JGK Memorial Research Library, Helsinki, Finland

**Keywords:** sudden infant death syndrome (SIDS), infection, diaphragm, pediatrics

## Abstract

Sudden infant death syndrome (SIDS) is the leading cause of death in infants between the ages of 1 and 12 months in developed countries. SIDS is by definition a diagnosis of exclusion, and its mechanism of action is unknown. The SIDS–Critical Diaphragm Failure (CDF) hypothesis postulates that the cause of death in SIDS is respiratory failure caused by CDF. Four principal risk factors contribute to CDF in young infants: undeveloped respiratory muscles, non-lethal infections, prone resting position, and REM sleep. Even relatively minor infections can cause an acute and significant reduction in diaphragm force generation capacity that in conjunction with other risk factors can precipitate CDF. CDF-induced acute muscle weakness leaves few, if any pathological marks on the affected tissue.Understanding the underlying mechanism of SIDS may help in formulating new approaches to child care that can help to further reduce the incidence of SIDS.

## Introduction

It has been recognized since antiquity that seemingly healthy infants can die suddenly and for no obvious reason during their sleep. Indeed, the Bible makes reference to such an incidence in the First Book of Kings. One of the first known medical autopsies of an apparent sudden infant death syndrome (SIDS) case was conducted by Samuel Fearn and published in the Lancet in 1834 (1). In his short communication, Fearn asked a simple question that has frustrated generations of doctors and researchers: ‘what was the cause of death?’ Over 175 years later the question is still unanswered (2). SIDS has been the target of an enormous research effort, with over 9,500 scientific articles published on the subject (3), but it remains one of the most mysterious disorders in medicine.

Our primary research interest, cancer cachexia (4), gave us a singular perspective into SIDS, and we formulated the SIDS–Critical Diaphragm Failure (CDF) hypothesis to answer Fearn's original question. It is perhaps surprising that research areas unrelated to SIDS could contribute meaningfully to the understanding of this complex syndrome, and yet there is a large body of experimental and clinical evidence that suggests that the cause of death in SIDS is respiratory failure caused by CDF. We argue that four factors contribute to CDF in SIDS: undeveloped respiratory muscles, non-lethal infections, prone resting position, and REM sleep. Infants younger than 6 months have undeveloped respiratory muscles that are susceptible to CDF, because even minor infections can precipitate a significant reduction in the diaphragm force generation capacity. The prone sleeping position significantly increases the diaphragm work-load, and the intercostal muscles that usually provide respiratory support are inactivated in REM sleep. The non-monotonic mortality rate of SIDS is explained by the passive maternal antibodies that effectively attenuate infections during the first month of life, and rapid development of the infant diaphragm that reaches the same fiber composition and pressure generation capacity as the adult muscle approximately 6 months *post partum*.

## SIDS

Research has identified over 40 potential SIDS risk factors (5), making the investigation of the syndrome extremely complicated. However, the salient and uncontentious characteristics of SIDS are well known; it affects fewer than 1 in 1000 live births in the developed world and is associated with sleep, a bell-shaped mortality incidence rate (peak age 2–4 months), premature and low-birth-weight infants, male gender (60% of cases), episodes of non-lethal infections, and the prone sleeping position (6–8). So far, no convincing genetic or congenital mechanism for SIDS has been identified.

SIDS is by definition a diagnosis of exclusion that has no identifiable cause of death. However, it is generally accepted that sudden death that is not caused by trauma must be due to either respiratory or circulatory failure (9). Clinical observations, heartrate, and respiratory recording of infants who subsequently succumb to SIDS and animal models strongly suggest a respiratory pathway for the syndrome (2).The absence of any obvious defects or abnormalities in the lungs or inspiratory muscles has led to the conclusion that respiratory failure in SIDS must be caused either by a dysfunctional control system (2,10) or upper airway obstruction (11). However, the dysfunctional respiratory control system hypothesis fails to explain many of the salient features of SIDS (6), and studies have found no significant respiratory abnormalities in infants who subsequently die of SIDS (12,13). Furthermore, both human and animal studies (14–17) suggest that upper airway occlusion is not a precipitating factor in SIDS, and obstructive apnea as the cause of SIDS is arguably doubtful.

The respiratory failure hypothesis is still favored by many SIDS researchers (11), but so far no convincing mechanism has been identified. Surprisingly, the possible role of the diaphragm in the etiology of SIDS has been largely ignored. In October 2010, there were only 41 PubMed hits with the search words ‘SIDS and diaphragm’ from a total of over 9,500 SIDS articles (18). Yet, the diaphragm is the main respiratory muscle, and as Muller and Bryan observe ‘respiratory failure is frequently respiratory muscle failure’ (19).

## Diaphragm and survival

Two muscles are essential for human survival, and while the vital role of the heart is obvious, the diaphragm is often overlooked. The diaphragm is a thin muscle that is shaped like a parachute and that separates the thoracic from the abdominal cavity. It is the main engine of the vital respiratory pump (20), and its contraction is critical for intrathoracic pressure generation (21). Anraku and Shargall note that ‘in adults, the diaphragm represents less than 0.5% of body-weight, but it is the most important muscle in the human body after the heart’. The incapacitation of supportive respiratory muscles, such as the intercostals, is not lethal, but bilateral diaphragm paralysis usually results in respiratory failure (22). The adult diaphragm can generate indefinitely approximately 40% of its maximum transdiaphragmatic pressure (Pdi_max_) (23), but it is susceptible to critical fatigue in certain conditions. Macklem succinctly observes that ‘the inspiratory muscles form a pump just as vital as the heart, and this pump can and does fail in much the same manner as the heart fails’ (20). In adults, respiratory muscle weakness is one of the most common causes of ventilatory failure (24).

We argue that the diaphragm is the locus minoris resistentiae in SIDS. The young infant has undeveloped secondary respiratory muscles, and the diaphragm is responsible for the majority of respiratory work (25). Assuming the same relative organ proportions as in the adult, a normal neonate weighing 3000 g would have a diaphragm that weighs approximately 12 g and is approximately 2 mm thick (26). Indeed, roughly similar dimensions are observed in preterm infants weighing between 1260 g and 2100 g who have diaphragms that are between 1.09 (±0.08) and 1.74 (±0.04) mm thick (27). An increase in diaphragm size correlates directly with inspiratory force (28), and full-term infants reach adult levels of Pdi_max_ at approximately 6 months of age (25). The diaphragm in young infants is also structurally immature and significantly more susceptible to fatigue than a fully developed muscle. A premature infant's diaphragm consists of only 10%fatigue-resistant type I fibers. The diaphragm of a full-term infant has 25% type I fibers, increasing to 40% at 3 months and to 50%–55% (adult share) (22) by the age of 7–8 months (19).The younger the infant, the smaller the oxidative capacity of the respiratory muscles and the higher the risk for ventilatory muscle fatigue (19). We suggest this may be a reason why low-birth-weight or premature infants have up to four times the risk of SIDS compared to full-term neonates (8). Overall, the respiratory muscles of the young infant appear to be poorly equipped to sustain increased work-loads. Muller and colleagues argue that ‘the normal preterm and term infant is very close to the threshold of diaphragmatic fatigue’ (29). Indeed, fatigue patterns can readily be observed if the work of breathing is increased in young infants (30). Still, some authors have suggested that the infant diaphragm is not at increased risk of fatigue and that it may be even more fatigue-resistant than the adult inspiratory muscle (31). We do not believe this contention is well supported by the existing evidence. However, even those who disagree about the relative fatigue risk of the infant diaphragm agreed that it can and does fatigue under certain conditions.

## Infection-induced acute diaphragm weakness

The central argument of the SIDS–CDF hypothesis is that even minor infections can cause a rapid and significant reduction in diaphragm strength. Respiratory muscle performance can decline for two reasons: due to a reduction in total muscle mass or due to a decline in muscle force generation capacity (32). The loss of muscle mass is an incremental process and is usually associated with chronic diseases such as cancer. The reduction of force generation capacity is associated with acute conditions such as sepsis where muscle strength can decline significantly within hours. We propose that an acute decline in muscle force-generating capacity is primarily responsible for CDF in SIDS cases. A large body of evidence strongly suggests that various types of infections, both viral and bacterial, can cause a significant and acute reduction in diaphragm force-generating capacity. Importantly, even minor infections can significantly reduce diaphragm strength without affecting muscle mass or histology.

Acute infections were shown to reduce significantly skeletal muscle strength in 1977 (33). Since then, numerous human and animal studies have shown that infections can induce severe diaphragm and secondary respiratory muscle weakness (34,35), whereas the cardiac muscle does not appear to be similarly vulnerable (36). Standardized endotoxins are often used to study diaphragm force reduction (37–39), but a broad range of pathogens, both viral and bacterial, has a similar effect. Human studies and animal models have shown that significant diaphragm weakness can be induced by endotoxin injection (40), bronchopulmonary infection (41), septic peritonitis (42), parasitic infection (43), and pathogens such as *Escherichia coli* (44), group B *Streptococcus* (45), *Streptococcus pneumoniae* (S) (46), *Pseudomonas aeruginosa* (41), *Bordetella pertussis* (47), *Phlebovirus* (48), as well as factors associated with upper respiratory tract infection (49). A case study of an infant with vulnerable respiratory muscles illustrates the point. The patient exhibited diaphragm muscle dystrophy as the sole anomaly and suffered from episodes of severe respiratory distress that required mechanical ventilation to sustain life (50). Interestingly, these episodes occurred only during *Staphylococcus aureus* or *Haemophilus influenzae* infections. The evidence cited above strongly suggests that various pathogens, both viral and bacterial in origin, can precipitate a significant reduction in the ventilatory capacity of the diaphragm.

It is well known that systemic infection can lead to severe loss of diaphragm strength and respiratory failure (51). However, also less severe infections can cause a significant reduction in the force-generating capacity of the diaphragm. Animal studies have shown that non-lethal sepsis induced by either *Streptococcus pneumoniae* or *E. coli* with no change in blood pressure, serum electrolytes, or acid status caused a significant impairment of diaphragm function (52). In human adults, Mier-Jedrzejowicz and co-workers observed already in 1988 that pulmonary function can deteriorate significantly (*P* < 0.05) after even apparently mild respiratory infections (53). In patients with vulnerable respiratory systems, routine infections have been shown to result in shortness of breath, reduction in vital capacity, and acute hypercapnia (49). Supinski and colleagues recently concluded that in vulnerable populations, ‘increasing evidence indicates that even minor infections can produce profound reductions in diaphragmatic force-generating capacity’ (54). A large body of experimental evidence supports this observation (34,41,43,55–58). It is important to note that the degree of diaphragmatic force reduction is determined not only by the intensity of the infectious assault but also by the immune and the developmental status of the host (41,45). Fundamentally, terms such as ‘lethal’ or ‘minor’ used to characterize infections are always relative.

Infections can reduce the force-generating capacity of the diaphragm by 50% in as little as 24 hours (54), and lead to severe respiratory muscle dysfunction and respiratory failure (45). In a study on human adults with routine upper respiratory tract infection, diaphragm strength fell significantly (*P* < 0.01), with the largest decline occurring between days 3 and 7 of clinical illness (53). Full recovery of respiratory pressure took place by day 14. Numerous other studies show that a significant reduction in diaphragm strength can occur within 1 to 48 h of infection indicating that a significant decline in the performance of the main respiratory muscle can occur very rapidly (34,41,42,49,51,59). However, infection-induced muscle force reduction is an acute but temporary event, and the diaphragm can recover its normal function relatively quickly. This may explain why near-SIDS cases do not manifest diaphragmatic weakness (60).

Animal models using either *Streptococcus pneumoniae* or *E. coli* endotoxin show that non-lethal sepsis impairs diaphragm function without affecting muscle mass or histology (46,61). Supinski and Callahan have observed that ‘24 h after administration of endotoxin, diaphragm specific force had fallen by 50% without any reduction in diaphragm muscle mass or diaphragm protein content, obvious depletion of major diaphragm protein bands or specific loss of myosin or actin stores’ (57). Sepsis reduces muscle strength long before contractile protein levels decline significantly (34). Others have reported similar results (62,63), confirming that even though infections can cause acute and severe diaphragm dysfunction, they leave few, if any pathological marks in the affected tissue. These findings are of some importance, because the absence of significant pathophysiological abnormalities in the inspiratory musculature of SIDS cases has led to the erroneous conclusion that the diaphragm does not play a role in the etiology of the syndrome (10).

The one pathological finding that characterizes over 70% of SIDS cases is intrathoracic petechiae (64).Goldwater reported recently that SIDS cases demonstrate intrathoracic petechial hemorrhages in the thymus (89.5%), pleura (80%), and epicardium (79.9%) (16). SIDS-related intrathoracic petechiae are not characteristic of asphyxia, strangulation (16), upper airway obstruction (17), or cardiac arrest (64). Rather, experimental evidence suggests that an infection together with gasping or a ‘struggle to breathe’ episode is required for the formation of characteristic intrathoracic petechial hemorrhages (17,65). Interestingly, the clinical signs of respiratory muscle fatigue are rapid shallow breathing, paradoxic chest wall/abdominal movements, and respiratory pauses combined with hyperventilation (20,66).

The data presented above strongly suggest that even minor infections can cause a significant and rapid reduction in diaphragm force-generating capacity without affecting muscle mass or histology. Surprisingly, the pathophysiological implications of infection-induced acute diaphragmatic weakness have not been previously considered in the context of SIDS even though it is well known that young infants have undeveloped and potentially vulnerable respiratory muscles.

## Mitochondrial dysfunction and the role of melatonin in SIDS

The molecular mechanisms behind infection-induced acute diaphragm weakness have been actively investigated. Systemic infection displays consistent similarities to the pathogenesis of SIDS (67), and can induce a significant (*P* < 0.001) reduction in the mitochondrial function (e.g. ATP-generating capacity) of the diaphragm muscle tissue (68). There is substantial evidence that excessive free-radical generation plays a central role in infection-induced myopathy and causes mitochondrial impairment. Infection causes significant changes in oxidative phosphorylation and induces the selective depletion of several electron transport chains in the respiratory muscles (32). Newborns and infants born prematurely are especially prone to oxidative stress because they are exposed to high oxygen concentrations and have reduced antioxidant defense mechanisms and high levels of free iron that are required for the Fenton reaction (69). Existing evidence strongly suggests that infections can produce rapid and profound alterations in the mitochondrial function of the diaphragm and severely disrupt its energy metabolism.

Melatonin (N-acetyl-5-methoxytryptamine) is a highly conserved small amphiphilic molecule that is biosynthesized from tryptophan. Serotonin is converted to melatonin in the pineal gland by arylalkylamine N-acetyltransferase (AA-NAT) and hydroxyindole O-methyltransferase (HIOMT) (70). Serotonin is a rate-limiting factor for melatonin. Melatonin has been shown to protect against oxidative stress in various divergent experimental systems (71). It is a highly effective antioxidant and free-radical scavenger that can significantly attenuate mitochondrial failure and preserve cell function and survival (72–74). In the clinical setting, melatonin has been shown to improve the clinical outcome in the septic newborn (75). Gitto and colleagues observe that ‘several clinical studies that used melatonin showed that it reduces oxidative stress in newborns with sepsis, distress or other conditions where there is excessive ROS/RNS (reactive oxygen species/reactive nitrogen species) production’(69). However, young infants exhibit transient melatonin deficiency for the first 2–4 months of life (76).

Pineal dysfunction and impaired melatonin metabolism have been associated with SIDS (77,78). Sturner and colleagues examined melatonin levels from the ventricular cerebrospinal fluid (CSF) in SIDS deaths compared to non-SIDS cases. After adjusting for age differences, melatonin levels were significantly (*P* < 0.05) lower among the SIDS infants (91 ± 29 pmol/L; *n* = 32) than among those dying of other causes (180 ± 27; *n* = 35) (79). Young infants who experienced a life-threatening event (ALTE) demonstrated significantly (*P* < 0.05) lower urinary excretion of the main melatonin metabolite 6-sulfatoxymelatonin compared to controls (1588 ng/24 h versus 3961 ng/24 h) (80). Serotonergic deficiency has repeatedly been associated with SIDS (81), suggesting that the role of the tryptophan–serotonin–melatonin pathway in the etiology of SIDS should be further investigated.

## The prone sleeping position and SIDS

A change in the recommended sleeping position for young infants from prone to supine has reduced SIDS deaths worldwide between 40% (Argentina) and 83% (Ireland) (2). SIDS can occur in the supine resting position, but any hypothesis regarding the etiology of SIDS must convincingly explain why the prone sleeping position is such a significant risk factor.

The effects of the prone sleeping position on diaphragm function have been considered previously (82). The hypothesis that the prone sleeping position significantly increases the work-load of the diaphragm was tested by Rehan and co-workers in a study with 16 healthy infants (83). The study showed that in the prone position the diaphragm is significantly thicker and, therefore, shorter at the end of both expiratory (EEV) and inspiratory lung volumes (EIV). The shortening of any muscle produces a marked fall in the maximal tension it can develop (19), indicating that the inspiratory muscle force of the diaphragm decreases as it shortens. Indeed, Rehan and colleagues note that ‘this degree of diaphragm shortening is similar to that seen with an increase in lung volume of 15%–30% of vital capacity’. Such increases in EEV can significantly impair diaphragm performance, and, as Rehan and colleagues conclude, ‘in adults, diaphragm strength and endurance as well as the efficiency of breathing are reduced by 40%–50% with this magnitude of increase in lung volume’. Greenough and colleagues have reported similar results in several clinical studies with premature infants and observe that respiratory muscle strength is significantly reduced in the prone compared to the supine position in (106–108).

Interestingly, an earlier study that measured infant breathing did not find significant differences in ventilatory performance as a function of sleep position (84). However, the study reported a significant increase (+66%) in rib-cage motion in the prone compared with the supine position. Others have observed that due to the high rib-cage compliance in young infants, the thorax motion increases either during deep inspiratory effort or when the intercostals are inactivated (31). As both the sleep position studies were conducted during non-REM (NREM) sleep when the intercostals are active, it is possible that the significantly increased thoracic motion in the prone position was due to increased diaphragm work needed to maintain ventilation pressure at the same level as in the supine position. Indeed, in another study with healthy infants sleeping in the prone position, chest wall distortion during REM sleep led to the reduction of the tidal volume, but ventilation was upheld by significantly (*P* < 0.01) increasing the work-load of the diaphragm (85). We suggest that healthy infants with normal diaphragms can easily tolerate the added mechanical strain that the prone position imposes on the diaphragm. However, for some infants the prone sleeping position combined with an infectious episode can increase the risk of CDF.

## Why is SIDS associated with sleep?

SIDS deaths often take place between midnight and 6 a.m. (5,86), and there is a close temporal relationship between SIDS and sleep (87). The original name for SIDS, ‘cot death’, reflects this basic observation. The reason for the preponderance of SIDS deaths during sleep is unclear, and while we should remember that newborns sleep up to 20 hours per day, there seems to be a causal link between sleep and SIDS. A premature infant spends up to 80% and a 3-month-old approximately 38% of his sleep in the REM phase (19). It is well known that the intercostals muscles show both phasic and tonic inhibition during REM sleep that renders them largely or totally inactive, and that this has a significant effect on respiratory function(31,88–90). The intercostals play a central role in stabilizing the thorax, and, as Davis and Bureau note, ‘chest wall muscles are critical for ventilation in the infant with a pliable chest wall’ (66). Others have suggested that the inactivation of the intercostals during REM sleep may be a contributing factor in SIDS (82). We argue that the inactivation of the intercostal muscles in combination with other factors can increase the risk of CDF.

The internal and external intercostal muscles are activated to attenuate diaphragm fatigue (19,91,92). In a study with preterm infants, diaphragm fatigue was followed either by a 50%–150% increase in intercostal activity or apnea that lasted 10–30 s and often required stimulation (93). In full-term infants, diaphragm fatigue provokes similar responses: the intercostal muscles are activated to support the respiratory effort or apnea lasting 5–20 s ensues (followed by intercostal muscle activation) (91). In another study, the diaphragmatic work-load in the young infant increased by over 150% (*P* < 0.001) during REM sleep (29). Diaphragmatic fatigue patterns were observed only in REM sleep together with marked reductions in intercostal activity and rib-cage retraction. In the young infant intercostal muscle support is even more important than in the adult because their rib-cage is mostly cartilaginous and significantly more compliant compared to adults. If the intercostals do not stabilize the rib-cage during inspiration, a significant portion of the power generated by the diaphragm will be wasted sucking in the ribs rather than fresh air (94), and to maintain the same tidal volume the work of the diaphragm has to increase substantially (88). This increased respiratory work-load may contribute to diaphragm fatigue.

The studies discussed above suggest that REM sleep is associated with increased diaphragmatic work-load and respiratory muscle fatigue in normal infants. Importantly, a fatigued diaphragm can be relieved only by decreasing the respiratory work-load or by activating the intercostals. If the critically fatigued muscle is not supported by the secondary respiratory muscles, ventilatory efficiency and muscle oxygenation will progressively decline and the risk of respiratory failure increases.

## The bell-shaped mortality rate in SIDS

One of the more puzzling aspects of SIDS is the bell-shaped mortality incidence rate. SIDS seems closely related to the developmental status of the infant, because 95% of the cases occur before the age of 6 months, and SIDS is rare in infants 1 year and older. Paradoxically, SIDS is also rare in infants younger than 1 month (5). SIDS clearly has a different type of mortality incidence rate compared to the monotonic distribution in congenital anomalies (6). Others have argued that bacterial toxins cause SIDS and that maternal antibodies can protect the infant during the first months of life (95). We suggest that the bell-shaped mortality distribution of SIDS is due to the passive immune protection maternal antibodies provide during the first month of life when the respiratory muscles are most vulnerable.

The age profile of SIDS deaths is reciprocal to infants' serum concentrations of immunoglobulin (96), and maternal antibodies can increase the immune resistance of neonates during the first months of life (97,98). Infants have varying levels of passive immune protection because the maternal antibody concentration determines the neonatal titer at birth (99). Full-term infants seem to have higher antibody titers than premature ones (100). However, the passive immune protection wanes relatively rapidly in all neonates. A study with 213 mother–infant pairs showed that the mean time to immunity loss for rubella and varicella was 2.1 months and 2.4 months, respectively (101). For measles the maternal antibodies endured for a median of 2.6 months (99). Maternal antibodies to the Varicellazoster virus (VZV) had a median half-life of 25.5 days in neonates (range 14.6–76.0 days) (102). Another study showed that 26% of infants had potentially protective antibody levels against pertussis (whooping cough) at delivery, but by week 6 nearly 90% had lost most of the original maternal antibodies (103). These and other studies (104,105) suggest that neonates can benefit from passive maternal immune factors for 1–2 months *post partum* but that the temporary immune protection wanes relatively quickly.

## Concluding remarks

We propose that the cause of death in SIDS is respiratory failure caused by CDF. It is well known that young infants have potentially vulnerable diaphragms and that infections can produce severe respiratory muscle dysfunction. We argue that ‘non-lethal’ infections are the main cause of CDF in SIDS. As such, there is no specific underlying genetic or congenital vulnerability in SIDS infants, and completely normal infants can die of SIDS. However, infants with weak or underdeveloped respiratory muscles who spend a large portion of the day in REM sleep and are exposed to infections are at increased risk. Dysfunctional melatonin metabolism may also be a contributing factor in SIDS. By definition, the infections associated with SIDS are not considered to be lethal, but they can cause a significant and acute loss of diaphragm strength. Additional stressors such as the prone sleeping position and REM sleep can move the infant closer to CDF and precipitate respiratory failure. Diagnosing CDF-precipitated respiratory failure is challenging because it usually does not affect muscle mass or histology.

An obvious question that the SIDS–CDF hypothesis might raise is that if young infants have vulnerable respiratory muscles and even minor infections can cause significant loss of diaphragm strength, why is SIDS not more common? Diaphragm fatigue is not the same as diaphragm failure, and while young infants are susceptible to CDF, they also have effective defense mechanisms that help preserve respiratory function. First, the immune system actively attenuates the negative effects of infectious assaults. Second, the activation of the intercostal muscles can decrease the respiratory burden and support a fatigued diaphragm. Third, the diaphragm develops rapidly, and by age 6 months has the same distribution of fatigue-resistant fibers and pressure-generating capacity as the adult muscle. Thus, moving from diaphragm fatigue to CDF usually requires a relatively rare combination of events that include an infectious episode, insufficient immune response, undeveloped respiratory muscles, continuous REM sleep, and often a prone resting position. Even if all these factors are present, the fatigued diaphragm may be rescued by altering the sleep state and activating the secondary respiratory muscles.

The clinical impact of an infection is dependent on its intensity as well as the immune and developmental status of the host. A potent infection can alone be lethal to a young infant, but a less severe infection will be dangerous only in conjunction with other factors. Yet, diagnosing an apparently ‘non-lethal’ infection as the cause of death is by definition a contradiction in terms. In a sense, a SIDS diagnosis reflects the failure to understand that apparently ‘minor’ infections can be fatal to some infants in certain circumstances. If the causes of infant mortality can be accurately ascribed, SIDS may no longer be needed as a diagnosis.This goal should be pursued in the spirit of Fearn's original question.
